# Fiber structures and material science in optical fiber magnetic field sensors

**DOI:** 10.1007/s12200-022-00037-0

**Published:** 2022-08-10

**Authors:** Jing Zhang, Chen Wang, Yunkang Chen, Yudiao Xiang, Tianye Huang, Perry Ping Shum, Zhichao Wu

**Affiliations:** 1 School of Mechanical Engineering and Electronic Information, China University of Geosciences (Wuhan), Wuhan, 430074 China; 2 Department of Electrical and Electronic Engineering, Southern University of Science and Technology, Shenzhen, 518055 China

**Keywords:** Optical fiber magnetic field sensors, Optical fiber structures, Magnetically sensitive materials, Optical fiber current sensors, Geomagnetic monitoring, Distributed magnetic fields sensors

## Abstract

Magnetic field sensing plays an important role in many fields of scientific research and engineering applications. Benefiting from the advantages of optical fibers, the optical fiber-based magnetic field sensors demonstrate characteristics of light weight, small size, remote controllability, reliable security, and wide dynamic ranges. This paper provides an overview of the basic principles, development, and applications of optical fiber magnetic field sensors. The sensing mechanisms of fiber grating, interferometric and evanescent field fiber are discussed in detail. Magnetic fluid materials, magneto-strictive materials, and magneto-optical materials used in optical fiber sensing systems are also introduced. The applications of optical fiber magnetic field sensors as current sensors, geomagnetic monitoring, and quasi-distributed magnetic sensors are presented. In addition, challenges and future development directions are analyzed.

**Graphical Abstract:**

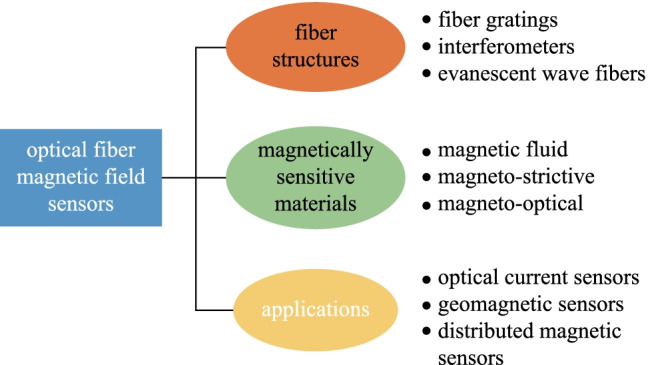

## Introduction

As technology advances, magnetic field sensors are playing an increasingly important role in scientific and industrial applications, including but not limited to electromagnetic system monitoring, geology exploration, biomedical detection, and aerospace engineering [1–4]. Traditional magnetometers based on the Hall effect, Overhauser effect, magneto-resistive effect, fluxgate, and other principles, have complex structures and high cost [2, 5]. Among the many available magnetic field sensors, optical fiber-based magnetic field sensors are gaining more and more attention as they offer unique advantages of light weight, small size, low cost, remote controllability, good security, and wide dynamic range characteristics [6, 7].

Traditional optical fibers are generally made of quartz glass or polymer, which are insulating materials and are not easily influenced by electric and magnetic signals. Therefore, optical fiber magnetic field sensors must be incorporated with specially designed optical structures and magnetically sensitive materials to modulate the physical properties (frequency, intensity, and phase) of light waves according to changes in external magnetic fields [8].

Among the numerous types of optical fiber magnetic field sensors, this paper intends to present sensors based on structured fibers with functional materials. The sensing structure and materials of optical fiber systems are discussed in detail. In addition, applications of optical fiber magnetic field sensors in optical current sensors, geomagnetic sensors, and quasi-distributed magnetic sensors are introduced. This work also analyzes the existing technical deficiencies and future development of optical fiber magnetic field sensors.

## Sensing structures of optical fibers

Obviously, fiber optical sensors based on insulating materials cannot be used directly for magnetic field sensing measurements. To achieve magnetic field sensing measurements, fiber optic magnetic field sensors must be combined with specially designed in-fiber optical structures that modulate physical properties such as the frequency, intensity, and phase of the light wave, and these changes are then observed and detected [8]. With the development of fiber optic sensing technologies and materials sciences, more and more fiber optic sensors are currently being developed for magnetic field sensing. This chapter will focus on the optical configurations of several common fiber optical magnetic sensors.

### Fiber grating based sensors

#### Fiber Bragg grating

**Fig. 1:**
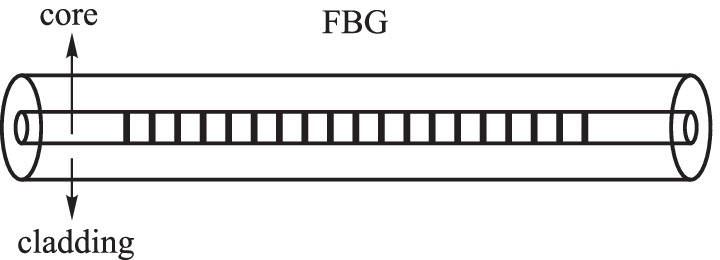
Schematic diagram of the fiber Bragg grating (FBG)

A common fiber Bragg grating (FBG) is a distributed Bragg reflector with a periodic distribution of refractive index modulation parts on the fiber core (Fig. 1), which can reflect light of selected wavelengths and transmits the others. FBGs can be fabricated by ultraviolet (UV) light, CO_2_ laser, and ultrafast laser processing. The periodicity of the refractive index modulation is at the submicron level and the reflected wavelength can be calculated by [9–12]

1 $$\begin{array}{c}{\lambda }_{\mathrm{B}}=2{n}_{\mathrm{eff}}\Lambda,\end{array}$$

where *λ*_B_ stands for the reflected Bragg wavelength, *n*_eff_ is the effective refractive index of the fiber core mode and Λ is the grating period. Typically, *n*_eff_ can be modulated by stresses and reductions in the diameter of the fiber cladding along the grating region, paving the way for magnetic field sensing.

**Fig. 2:**
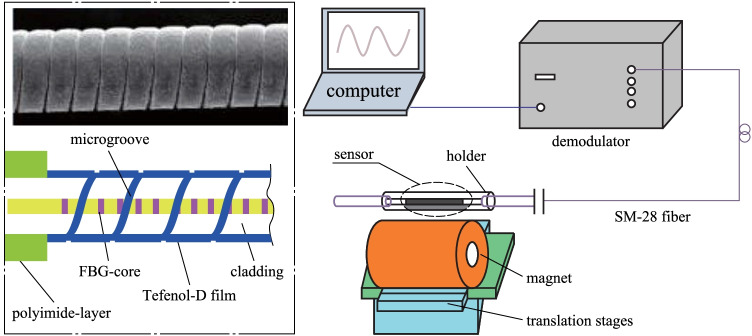
Sputter fiber Bragg grating (FBG) sensor structure [14]. Copyright 2013, Dai Y. Reproduction with permission

In 2009, Minghong Yang et al*.* deposited a magneto-strictive thin TbDyFe film on cladding etched FBGs for magnetic field sensing by magnetron sputtering process. As the magnetic field intensity increased, the TbDyFe film deformed, causing a change in the grating period of the FBG and thus introducing a wavelength shift. Sensing experiments were carried out on FBGs with 0.8 μm TbDyFe coating in the magnetic field intensity range of 0–50 mT, and different diameters of the FBG was considered. The sensitivity of a FBG with a diameter of 125 μm was 0.386 pm/mT, that of FBG with a diameter of 105 μm was 0.563 pm/mT and that of a FBG with a diameter of 85 μm was 0.95 pm/mT [13]. In 2013, Yutang Dai et al*.* proposed a magnetic field sensor based on a Terfenol-D coated FBG with a spiral microstructure. As shown in Fig. 2, a femtosecond laser processing system was used to write the spiral microstructure into the FBG cladding, which made the sensitivity of this magnetic field sensor about 5 times higher than that of a non-spiral structured FBG. In the magnetic field intensity range of 0–140 mT, the sensitivity was about 0.7 pm/mT [14]. In 2018, Xinyong Dong et al*.* proposed a magnetic field sensor based on the magnetic fluid -infiltrated phase-shifted FBG. This phase-shifted FBG consisted of two FBG sections with a micrometer-level gap between them. The refractive index of the magnetic fluid varied with the magnetic field and entered the sensing structure through the gap, thus affecting the transmission spectrum. The sensitivity was 2.42 pm/Oe for magnetic field intensity in the range of 0–120 Oe [15].

#### Long-period fiber grating

**Fig. 3:**
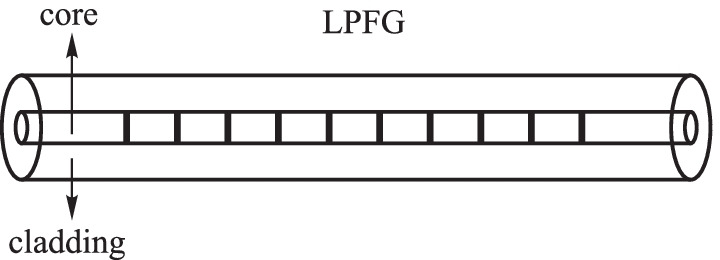
Schematic diagram of the long-period fiber gratings (LPFG)

The period of refractive index modulation in long-period fiber gratings (LPFG) is much larger than the wavelength of light wave (Fig. 3) [16]. LPFG works based on the coupling effect of the co-directionally propagated core mode and cladding mode that satisfies the phase-matching condition and generates a series of loss peaks at the corresponding resonant wavelengths in the transmission spectrum. The shift of the LPFG resonance peak can be modified by the change in the effective refractive index of the different cladding modes as shown by

2 $$\begin{array}{c}{\lambda }_{i}=\left({n}_{\mathrm{core}}-{n}_{\mathrm{clad}}^{i}\right){\Lambda }_{\mathrm{LPFG}},\end{array}$$

where *λ*_*i*_ represents the central wavelength of the *i*th attenuation band, and *n*_core_ and ${{n}_{\mathrm{clad}}^{i}}$ are the effective refractive indices of the core mode and the *i*th cladding mode, respectively. Λ_LPFG_ represents the grating period. Similar to FBG, the resonant peak shifting of LPFG is also widely used in magnetic field sensing.

In 2012, Tao Zhu et al. designed a fiber optic magnetic field sensor based on a D-type long-period fiber grating and an aqueous magnetic fluid. The LPFG had a period of 685 µm and it achieved a sensitivity of 176.4 pm/mT when the magnetic field intensity increased to 189.7 mT [17]. In 2014, Chia-Chin Chiang and Zheng-Jie Chen proposed the use of an electroforming long-period optical fiber grating (ELPFG) with a periodic polymer-metal (SU-8 photoresist and nickel) structure. The experimental results showed that in the magnetic field intensity range from 0 to 47.6 mT, the sensitivity of the LPFG for grating period of 620 μm was 0.045 nm/mT, the LPFG sensitivity for 630 μm grating period was 0.161 nm/mT, and the LPFG sensitivity for 640 μm grating period was 0.148 nm/mT [18]. In 2021, Shen Liu and Jianqing Li et al*.* reported a helical long-period fiber grating (HLPFG) based on the three-core fiber (TCF) for magnetic field sensing. The TCF-HLPFG was combined with a U-shaped aluminum wire and placed in a perpendicular magnetic field. The electrical current flowing through the aluminum wire generated Ampere force, which could bend the TCF-HLPFG and cause shifting of the resonator peaks. The sensitivity of this device was measured to be 456.5 pm/mT in the magnetic field intensity range of −15 to 15 mT [19].

#### Tilted fiber Bragg grating

**Fig. 4:**
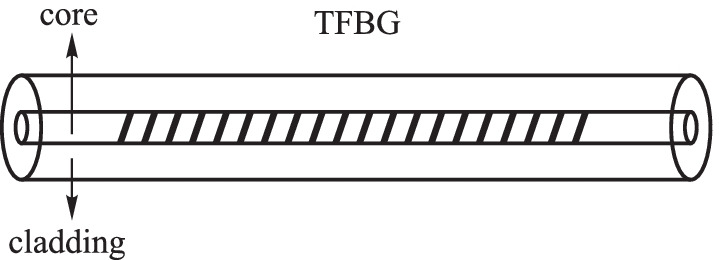
Schematic diagram of the tilted fiber Bragg grating (TFBG)

With the development of optical fiber technology, the tilted fiber Bragg grating (TFBG) with periodic refractive index modulation along the fiber core has been created (Fig. 4) [20]. The unique design in the device is a certain inclination angle between the grating plane of TFBG and the fiber cross-section. The consequent introduction of an angle between the fiber axis and the wave vector direction in TFBG leads to fiber core mode coupling into the cladding and finally dissipating in the cladding. The resonance wavelength of the core mode (*λ*_TFBG_) and the *i*th cladding mode (${{\lambda}_{\mathrm{clad}}^{i}}$) are determined by the phase-matching condition, which can be expressed as [21, 22]

3 $$\begin{array}{c}{\lambda }_{\mathrm{TFBG}}=\frac{2{n}_{\mathrm{eff},\mathrm{core}}\Lambda }{\mathrm{cos}{\theta }_{\mathrm{TFBG}}},\end{array}$$

4 $$\begin{array}{c}{\lambda }_{\mathrm{clad}}^{i}=\frac{\left({n}_{\mathrm{eff},\mathrm{core}}^{i}+{n}_{\mathrm{eff},\mathrm{clad}}^{i}\right)\Lambda }{\mathrm{cos}{\theta }_{\mathrm{TFBG}}},\end{array}$$

where *n*_eff,core_ is the refractive index of the core mode at *λ*_TFBG_. ${n}_{\mathrm{eff}, \, \mathrm{core}}^{i}$ and ${n}_{\mathrm{eff}, \, \mathrm{clad}}^{i}$ are the refractive indices of the core mode and the *i*th cladding mode at ${{\lambda}_{\mathrm{clad}}^{i}}$, respectively. *θ*_TFBG_ is the tilt angle between the grating planes and the fiber axis. Λ corresponds to the nominal grating period and Λ_TFBG_ represents the grating period along the axis fiber, which can be expressed as Λ_TFBG_ = Λ/cos*θ*_TFBG_.

In 2011, Paul Childs et al*.* designed a cladding ring sensor with ferrofluid surrounding the cladding consisting of two identical TFBGs spaced at a fixed separation. The sensing principle of the device was based on the reduction of the fringe visibility of the interference in the ghost mode. In the magnetic field intensity range of 0.03 to 0.14 T, the sensitivity was 0.4 nm/T [23]. In 2013, Xinyong Dong et al. proposed a magnetic field sensor composed of a TFBG coated by magnetic fluid and cascaded by a chirped-FBG (CFBG). Transmission of the TFBG was modulated by the changeable refractive index of the magnetic fluid and CFBG reflected broadband of light spectrally located at the cladding mode resonances region of the TFBG. When the magnetic induction strength was increased from 0 to 140 Gs, the sensitivity of this sensor could reach 147 nW/Gs [24].

### Fiber interferometric sensors

#### Mach–Zehnder interferometer

**Fig. 5:**
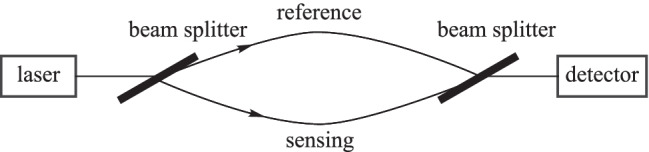
Schematic diagram of the Mach–Zehnder interferometer (MZI)

A fiber-based Mach–Zehnder interferometer (MZI) is able to detect the relative phase shift variation between two optical paths caused by changes in environmental physical parameters, such as the magnetic field. Therefore, the two optical paths can be used as one reference path and one sensing path (Fig. 5). Note, if the MZI consists of only one optical fiber, the phase shift difference can be induced by the effective refractive index difference between the fiber core and the cladding, which leads to mode dispersion of the optical signal [25–29].

**Fig. 6:**
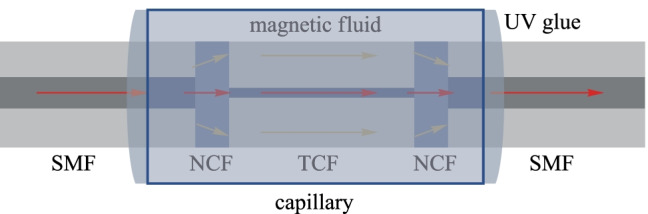
Magnetic fluid sensor based on the no-core-fiber/thin-core-fiber/no-core-fiber (NTN) structure [30]. Copyright 2022, Elsevier B.V. Reproduction with permission

In 2022, Xiaoyan Sun et al*.* proposed an intensity-modulated magnetic field sensor based on MZI. This MZI consists of a no-core-fiber/thin-core-fiber/no-core-fiber (NTN) structure with a hydrofluoric acid solution etched fiber cladding. A diagram of this sensing structure is shown in Fig. 6. In the magnetic field intensity range of 0 to 55 Oe, the sensor achieved a sensitivity of 0.418 dB/Oe with the etching time of 180 s and 0.563 dB/Oe with the etching time of 270 s [30].

#### Fabry−Perot interferometer

**Fig. 7:**
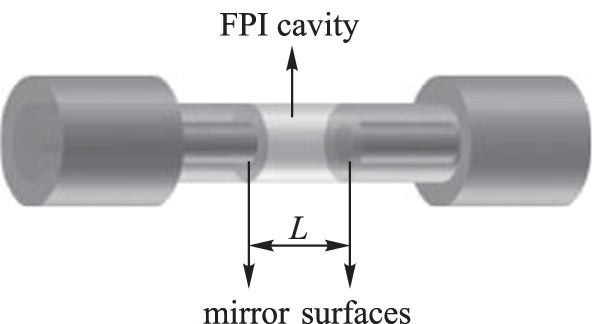
Schematic representation of an Fabry−Perot interferometer (FPI)

The Fabry−Perot interferometer (FPI) is a multi-beam interferometer consisting of two parallel reflecting surfaces, forming a cavity of a given intermediate medium between the two reflecting surfaces, as shown in Fig. 7 [7, 31]. FPIs are very sensitive to perturbations that affect the interior of the cavity. The basic principle of the FPI is based on the superposition of multiple reflections of light between two reflective plates within the cavity.

By controlling the distances between the two reflective surfaces or the refractive index of the intermediate medium, different transmission spectra can be obtained at the output due to the correlation between the spectral modulation period Λ_FPI_ and the refractive index and physical length of the cavity, as described by

5 $$\begin{array}{c}{\Lambda }_{\mathrm{FPI}}=\frac{{\lambda }^{2}}{2n{L}_{\mathrm{FPI}}}.\end{array}$$

The reflecting surface at the rear end produces a periodic reflection in the spectral frequency domain, and the phase of the reflected light signal is given by [32]

6 $$\begin{array}{c}{\mathrm{\varnothing }}_{\mathrm{FPI}}=\frac{4\uppi }{\lambda }n{L}_{\mathrm{FPI}},\end{array}$$

where *λ* is the wavelength of the input optical signal, *n* represents the refractive index of the intermediate medium material in the cavity, and *L*_FPI_ is the length of the FPI cavity.

**Fig. 8:**
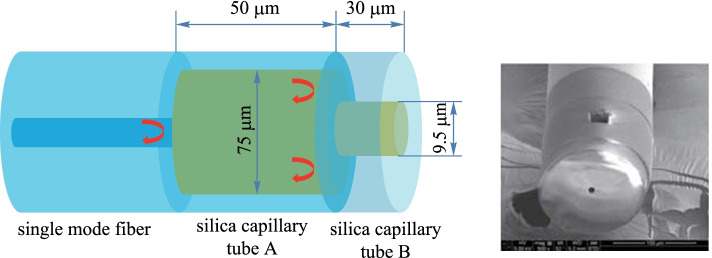
Open cavity Fabry−Perot interferometer based on optical fiber for magnetized field sensing [36]. Copyright 2017, IEEE Xplore. Reproduction with permission

For magnetic field sensing applications, researchers usually fill the magnetic-sensitive material in the fiber-based FP air cavity. Upon the introduction of a magnetic field, the refractive index of the magnetic material changes, resulting in changes of the interference wavelength [33–35]. In 2017, Yangzi Zheng et al*.* proposed and implemented a compact fiber-optical FP sensor with an embedded microfluidic channel for static magnetic field measurement (Fig. 8). The magnetic fluid penetrated the cavity through the micro slot on the FP cavity. The system realized magnetic field sensitivity of 418.7 pm/Oe [36].

In 2021, Yong Zhao et al*.* proposed a magnetic field measurement method based on the magnetic volume effect, where magnetic fluid was filled into a hollow fiber. When the magnetic induction strength ranged from 109.6 to 125.8 Gs, the sensitivity of the magnetic field sensor was − 4219.15 pm/Gs [37].

#### Sagnac interferometer and Michelson interferometer

**Fig. 9:**
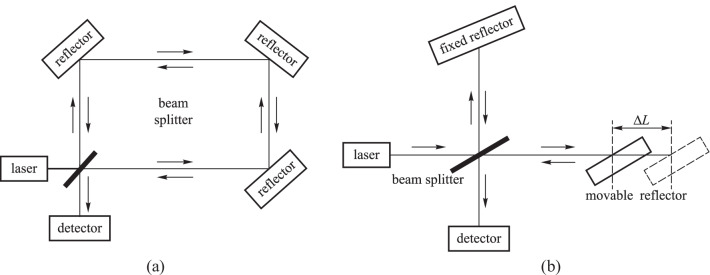
**a** Schematic diagram of the Sagnac interferometer. **b** Schematic diagram of the Michelson interferometer

A Sagnac interferometer (SI) works by the Sagnac effect. The principle is shown in Fig. 9a. The beam from the light source is split into two beams by a splitter that produces the same optical paths but in opposite directions. When the interferometer system is affected by the surrounding environment, such as a magnetic field, the optical paths of the two beams become no longer equal, and the phases of light waves in the two beams become different, resulting in the changes in interference fringes and transmission spectra as well [38–41].

Similar to the SI, the Michelson interferometer (MI) also divides the beam from the light source into two beams but with a phase difference of 180º. As shown in Fig. 9b, one beam is directed to the fixed reflector, and another beam is directed to the movable reflector. A magnetic field can influence the position of the movable reflector, resulting in an optical path difference and changes in the interference pattern [42–44].

In 2017, Shengli Pu et al*.* designed a microfiber Sagnac loop (MSL) for magnetic field sensing with the sensitivity of 19.4 pm/Oe, in which the microfiber coupling regions were coated with magnetic fluids [45]. In 2021, Xinxing Feng, Yi Jiang, and Han Zhang proposed a magnetic field sensor based on the MI composed of a 3 × 3 coupler, where the magnetic field transducer acted as a mechanically amplified structure, and the deformation generated by a TbDyFe rod under the action of a magnetic field was amplified through a transducer and transmitted to a polarization-preserving fiber, resulting in a change in the length of the fiber. The sensing sensitivity of 0.4471 V/μT over a magnetic field intensity range of 1 to 8 μT was achieved [46].

### Evanescent field based sensors

#### Tapered fibers

**Fig. 10:**
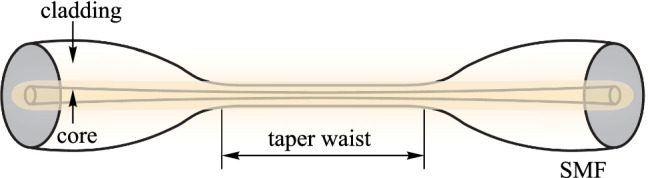
Schematic diagram of a tapered fiber

To expose the evanescent field along optical fibers, the fiber diameter needs to be reduced. After removing the coating layer, the optical fiber can be heated to the material’s molten state, and be stretched at both ends, elongating and thinning the original fiber (Fig. 10) [47]. The light leaks out from the taper waist and forms the evanescent field, which interacts with the surrounding environment [48–51].

Benefiting from the structure of tapered microfibers, in 2021, Yu Zhang et al*.* proposed an optical fiber sensor built with spider silk for magnetic field measurement. Spider silk with magnetic nanoparticles was evenly wound in the taper waist and combined. In the magnetic field intensity range of 0–120 Oe, the sensitivity of the sensor reached 1126.3 pm/Oe [52].

#### D-shaped fibers

**Fig. 11:**
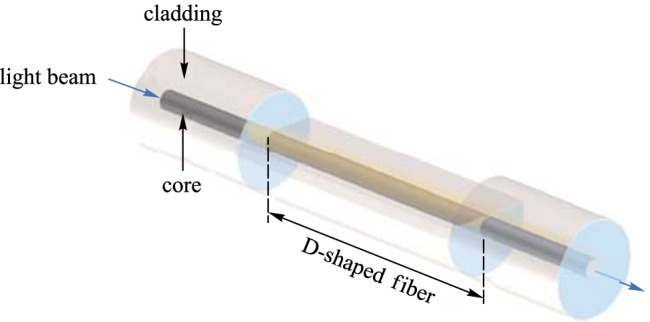
Schematic diagram of an etched fiber

Etched or polished D-shaped optical fibers are also widely used in evanescent field-based sensors. As shown in Fig. 11, the fiber is chemically etched or mechanically polished to remove the upper part of the fiber cladding and to expose the fiber core [53]. With this structure, the evanescent field can be exposed to the environment and thus achieve environmental sensing [54–57].

In 2018, Deqiang Cheng et al. proposed a surface plasmon resonance (SPR) magnetic field sensor based on D-type optical fibers and magnetic fluid-infiltrated photonic crystal fibers (PCFs). By using a combination of SPR and directional resonance coupling in the PCF, a two-parameter matrix approach was used to eliminate the temperature-induced error. Conventional gold-layered composites were replaced by Au–Ag films to reduce losses. A sensing sensitivity of 0.87 nm/mT was obtained over a magnetic field intensity range of 0 to 55 mT [58].

#### U-shaped fibers

**Fig. 12:**
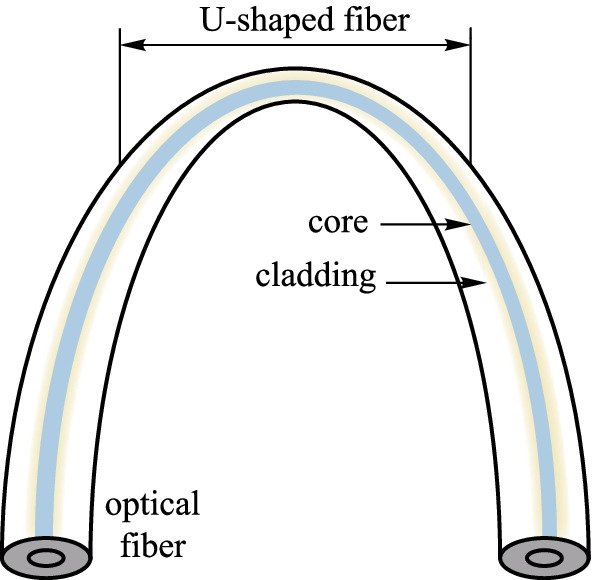
Schematic diagram of the U-shaped fiber

As shown in Fig. 12, the U-shaped optical fiber also can expose the evanescent fields of optical fibers [59]. The U-shaped fiber is achieved by bending the fiber to change the total reflection conditions in the fiber, obtaining the exposed evanescent field at the bending region [60]. In 2021, Qijing Lin et al*.* designed a magnetic field sensor based on U-shaped optical fibers. The fibers were placed into a cylindrical container filled with magnetic fluid. In the magnetic field intensity range of 0–10 mT, the resonant wavelengths were red-shifted and blue-shifted when the magnetic field was applied perpendicularly and parallel to the sensor, respectively. This U-shaped fiber had sensitivities of 0.78 and 0.12 nm/mT when the magnetic field was parallel and perpendicular to the sensor, respectively [61].

In this section, we introduced a variety of optical structures, such as fiber gratings, optical interferometers, and evanescent field fibers, which can modulate the light propagation properties according to the environmental characteristics for fiber-based magnetic field sensing. Table 1 summarizes some typical optical fiber magnetic fiber sensors according to the classification of optical configurations reported in this section. The different magnetic sensitive substances, the sensing sensitivities, and the magnetic field measurement ranges are also listed.

**Table 1:** Performance parameters of fiber optic magnetic field sensors

Sensing structure	Sensing materials		Sensitivity/resolution^a^	Measurement range^a^	Reference
FBG	MS	TbDyFe	0.950 pm/mT	0–50 mT	[13]
		Terfenol-D	0.7 pm/mT	0–140 mT	[14]
			3.7102 pm/mT	0–183.38 mT	[11]
	MF	EMG 605	24.2 pm/mT	0–12 mT	[15]
	MO		7.9618 pm/mT		[12]
LPFG	MF	EMG 605	176.4 pm/mT	0–189.7 mT	[17]
	Aluminum wires		456.5 pm/mT	− 15 to 15 mT	[19]
TFBG	MF	EMG 605	0.4 pm/mT	30–140 mT	[23]
			1470 nW/mT	0–14 mT	[24]
MZI	MF	Fe_3_O_4_	2.08 × 10^4^ nm/mT	5–9.5 mT	[27]
		Ni Fe_2_O_4_	66.7 dB/mT	0.75–2.2 mT	[28]
	MS	GMM	1.33 MHz/mT	20.9–58 mT	[29]
FP	MF	EMG 905	431 pm/mT	0–40 mT	[33]
		EMG 605	530 pm/mT	0–70 mT	[34]
		EMG 607	4187 pm/mT	0.12–15.341 mT	[36]
		Fe_3_O_4_	1540 pm/mT	1–8 mT	[35]
			−4 .21915 × 10^4^ pm/mT	10.96–12.58 mT	[37]
	MO	La_0.7_Ba_0.3_MnO_3_	228 pm/mT	0–20 mT	[31]
SI	MF	EMG 605	5928 pm/mT		[40]
		Ferrofluid	73 pm/mT	10–40 mT	[41]
MI	MF	Fe_3_O_4_	64.9 pm/mT	0–120 mT	[43]
	MS	TbDyFe	36 V/mT	0–0.1 mT	[44]
			447.1 V/mT	0–0.008 mT	[46]
Tapered fibers	MF	EMG 607	1 × 10^4^ pm/mT	0–10 mT	[49]
		Ferromagnetic nanoparticles	719.8 pm/mT	4–12 mT	[51]
	MS	Magnetic tape	70 pm/mT	15–60 mT	[50]
		Modified silk	1.1263 × 10^4^ pm/mT	5.5–8.5 mT	[52]
D-shaped fiber	MF	Fe_3_O_4_	− 168.6 pm/mT	12–32.5 mT	[55]
		EMG507	1.256 mT/μs	0–65 mT	[56]
		MnFe_2_O_4_	6.9 × 10^4^ pm/mT	7.5–12.5 mT	[57]
U-shaped fiber	MF	Fe_3_O_4_	870 pm/mT	0–55 mT	[58]
		EMG605	3185.2 pm/mT	1.6–9.6 mT	[60]
		Fe_3_O_4_	780 pm/mT (Parallel) 120 pm/mT (Perpendicular)	0–10 mT	[61]

^a^The data units have been converted, and the conversion relationships are as follows: 1 T = 1000 mT, 1 mT = 10 Gs, 1 mT = 10 Oe, and 1 Oe = 1000/(4π) A/m

Note  that there are two systems of units for magnetic field intensity, the International System of Units and the Gauss System of Units. In the International System of Units, the unit of magnetic field intensity is ampere per meter (A/m) and the unit of magnetic induction strength is the Tesla (T). In the Gauss System of Units, the unit of magnetic field intensity is the oersted (Oe) and the unit of magnetic induction intensity is the Gauss (Gs). The units of magnetic field sensitivity in the text can be calculated by converting the above-mentioned relation [62].

## Magnetically sensitive materials for optical fiber sensing systems

“Magnetically sensitive materials” is the collective term for materials that are sensitive to magnetic fields and serve to convert magnetic signals into other kinds of signals. Many types of magnetically sensitive materials are used to modulate the shape or refractive index of fiber according to the intensity and direction of the magnetic field, as one of the key units in optical fiber magnetic field sensors. In general, they can be divided into the following three categories: magnetic fluid (MF) materials, magneto-strictive (MS) materials, and magneto-optical (MO) materials.

### Magnetic-fluid materials

#### Principle of magnetic fluids

**Fig. 13:**
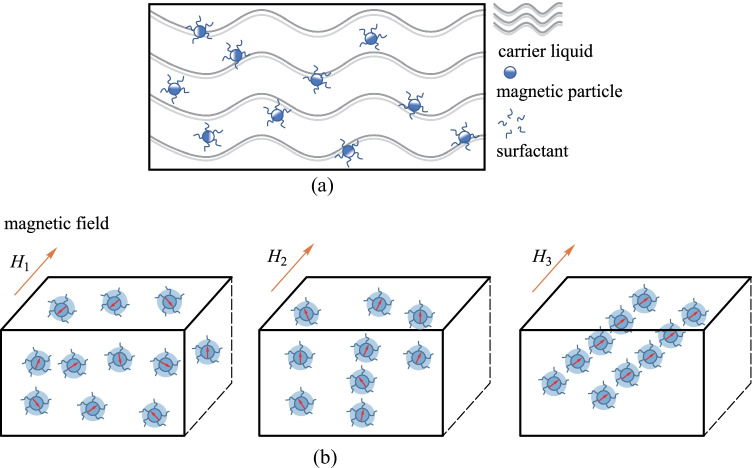
**a** Schematic diagram of the composition of magnetic fluid. **b** Variation of magnetic fluid nanoparticles under different magnetic field intensities (magnetic field intensity: *H*_3_ > *H*_2_ > *H*_1_)

In recent decades, studies of magnetic fluids (MFs) have rapidly developed in the field of fiber optic sensing. As shown in Fig. 13a, a magnetic fluid is a stable colloidal system with nanoscale magnetic particles (i.e., γ-Fe_2_O_3_ [63], Fe_3_O_4_ [64], α-Fe_3_N [65]) being encapsulated by surfactants. The particles are uniformly dispersed in a carrier fluid. In addition, magnetic fluids have many optic properties, including the tunable refractive index, birefringence, and Faraday Effect behavior [66, 67]. When a magnetic fluid is exposed to an external magnetic field, the structural pattern of the magnetic particles in the magnetic fluid changes with the intensity and direction of the magnetic field. Specifically, the magnetic particles tend to aggregate into chains along the magnetic field direction (Fig. 13b).

#### Magnetic field sensor based on magnetic fluid materials

Currently, many fiber-based magnetic field sensors are designed based on the tunable refractive index property of magnetic fluids. Yu Ying et al*.* proposed a magnetic field sensor combining D-shape optical fiber and magnetic fluid grating. Their simulation results showed that a maximum sensitivity of 6.9 nm/Oe can be achieved [57]. Tonglei Cheng et al*.* proposed a magnetic field sensing system based on the surface plasmon resonance. In this sensor, MF was contained in a capillary tube made of epoxy resin. The sensor sensitivity reached 303 pm/Gs (3030 pm/mT) in the study [68]. Nunzio Cennamo et al*.* combined the D-shape plastic optical fiber with a magnetic fluid. The sensitivity of this sensor was about 6800 pm/mT and the resolution was about 0.029 mT [69]. Yong Zhao et al*.* proposed a magnetic field sensing structure based on the magnetic-volume effect, which significantly enhanced the sensitivity of the sensor [37]. Chunfu Cheng et al*.* proposed a sensor based on the frequency-shifted interferometry fiber loop ringdown (FSI-FLRD) and the magnetic fluid, which provided a practical way for quasi-distributed magnetic field sensing. The sensitivities of the two sensing channels were − 9.38 × 10^–5^/(km·Oe) and − 2.28 × 10^–5^/(km·Oe), respectively [70].

### Magneto-strictive materials

#### Principle of magneto-strictive effect

**Fig. 14:**
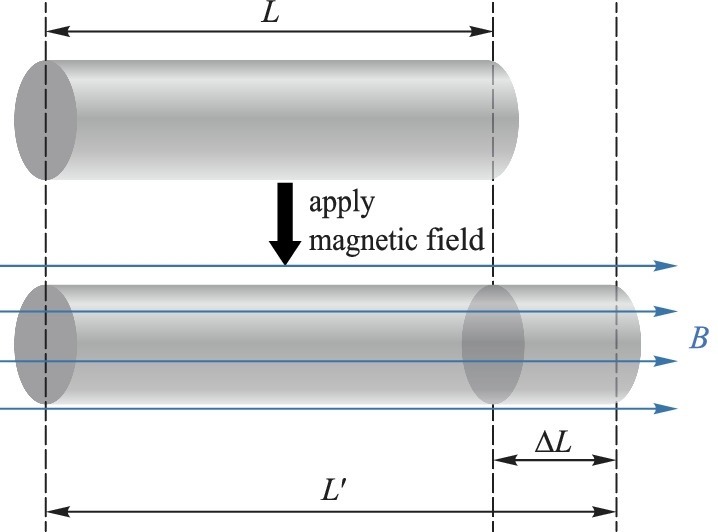
Principle of magneto-strictive effect

Magneto-striction is a special feature of ferromagnets and relates to the change in the shape of the ferromagnets due to the change in its magnetization state under an applied magnetic field. The dimensional change of the ferromagnets is dependent on the magnitude of the magnetic field [71, 72]. The magneto-strictive effect mainly affects the variation of the length of ferromagnets according to the magnetic flux density, *B* (Fig. 14). The linear magnetostriction coefficient *λ*_MS-coff_ can be calculated from the length and elongation of the sample with

7 $$\begin{array}{c}{\lambda }_{{\text{MS-coff}}}=\frac{\delta L}{L}, \end{array}$$

where *L* is the length of the sample and *δL* is the amount of variation in the length of the sample. When the temperature is constant, *λ*_MS-coff_ increases with the magnetic flux density, *B*, until it reaches the saturation value *λ*_s_, which is named the saturation magnetostriction coefficient.

#### Magnetic field sensor based on magneto-strictive materials

**Fig. 15:**
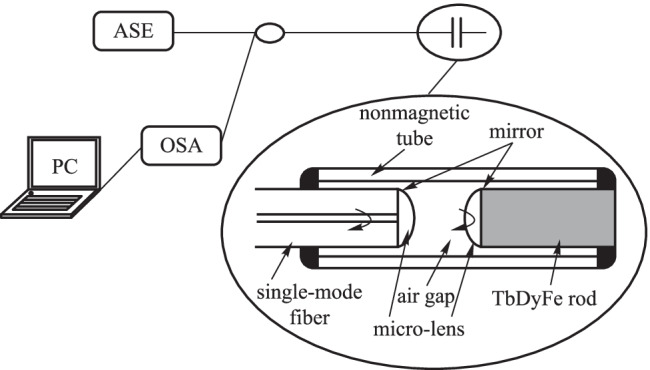
Experimental setup of the high-finesse extrinsic Fabry–Perot interferometers (EFPI) based fiber-optic magnetic-field sensor [75]. Copyright 2015, Elsevier B.V. Reproduction with permission

Terfenol-D is one of the most promising magneto-strictive materials with the chemical formula Tb_0.27_Dy_0.73_Fe_1.95_ [73, 74]. Minghong Yang et al*.* proposed to deposit magneto-strictive TbDyFe thin films on cladding-etched FBGs [13]. Yi Jiang et al*.* used the TbDyFe material in a Fabry–Perot cavity, which is shown in Fig. 15 [75]. The sensor demonstrated a high sensitivity of 1510 nm/mT and a magnetic resolution of 25 nT [75]. Andrea Cusano et al*.* investigated a fiber optic triaxial magnetic field sensor based on the fiber Bragg gratings and the super magneto-strictive material Terfenol-D, which can be applied to magnetic resonance imaging [76].

The encapsulation of Terfenol-D particles with resin polymers has led to a significant improvement in the operating performance of the composites in high-frequency conditions [77]. Siu Wing Or et al*.* proposed a novel sensor that used an epoxy-bonded Terfenol-D particle pseudo-1-3 magnetostrictive composite (MC) as the magnetically driven material and FBG as a strain sensor. The sensor has a quasi-static peak wavelength shift sensitivity of 4.66 × 10^–3^ nm/kA/m and a quasi-static magneto-strictive strain sensitivity of about 3.4 ppm/kA/m [11].

Recently, Muguang Wang et al*.* have proposed a magnetic field sensing scheme with a large measurement range using an optoelectronic oscillator and a Mach–Zehnder interferometer (MZI). Super magneto-strictive material was used for the magnetic field sensor. The sensing system had a magnetic field sensitivity of 1.33 MHz/mT and a magnetic field intensity measurement range of 20.9–58 mT, and the temperature-induced interference error is only 0.09 mT [29].

### Magneto-optical materials

#### Magneto-optical effect

**Fig. 16:**
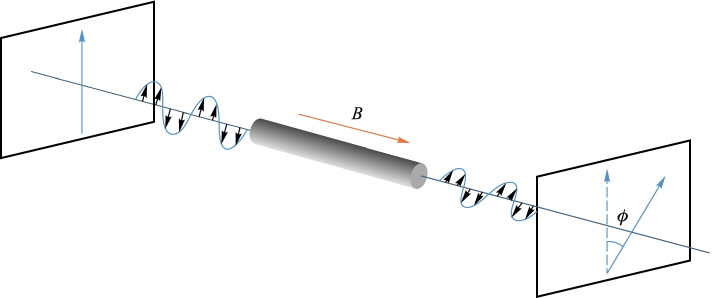
Principle of Faraday magneto-optical effect

The most used magneto-optical effect in fiber sensing systems is the magneto-optical Faraday effect. As shown in Fig. 16, when a beam of linearly polarized light passes through a magneto-optical material, the magnetic field applied along the light propagation direction causes the polarization plane of the linearly polarized light to rotate, and the rotation angle is called the Faraday rotation angle *Ф* given by [78]

8 $$\begin{array}{c}\phi =V{\int }_{0}^{L}B\mathrm{d}L=VLB,\end{array}$$

where *L* is the distance that light travels through the material and *B* is the component of external magnetic flux density in the direction of light propagation. *V* is the Verdet constant, which can be used to characterize the intensity of the ability to undergo Faraday rotation in magneto-optical crystals. The magnitude of the Verdet constant depends on the properties of the medium, the ambient temperature, and the wavelength of the incident light. The Verdet constants are very small in most substances, but glasses doped with rare-earth ions have larger Verdet constants [79].

#### Magnetic field sensor based on magneto-optical materials

Since the discovery of the Faraday magneto-optical effect, research on Faraday magneto-optical materials has been conducted for half a century, and magneto-optical materials with superior performance have been the focus of attention. In recent years, magneto-optical materials based on garnet crystals have been widely studied. Common magneto-optical crystals mainly include yttrium iron garnet [80], terbium gallium garnet [81], terbium aluminum garnet [82], and other rare earth doped garnet.

Junjie Jiang et al*.* proposed a magneto-optical fiber optic sensor based on yttrium iron garnet (YIG) with a magnetic field intensity measurement range of 0–24 mT. This sensor could be used to measure the magnetic field of high-temperature superconducting coils in liquid nitrogen [83]. M. K. Virchenko et al*.* used the magneto-optical effect of iron garnet to control the displacement and tilt angle of the valve adjustment element [84].

**Fig. 17:**
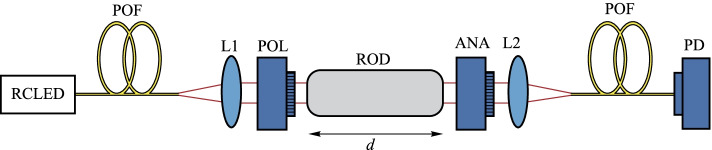
Schematic representation of the optical head of the current sensor [88]. Copyright 2013, Zubia J. Reproduction with permission

Min Huang’s group proposed a photocurrent/magnetic field sensor based on cerium-substituted yttrium-iron garnet single crystals. The new sensor had a 31.1-fold increase in sensitivity as compared to the pure YIG sensor [85]. Richard Forber et al*.* fabricated an ultra-small magnetic field sensor using bismuth-doped rare earth iron garnet (Bi:RIG), which can detect magnetic field intensity as low as 11 A/m [86]. Joaquim F. Martins-Filho et al*.* proposed a sensor based on cerium-doped yttrium iron garnet (Ce:YIG) with a dynamic magnetic field intensity sensing range up to 0.2 T [87]. In addition to these magneto-optical crystals, Joseba Zubia et al*.* developed a new low-cost optical current sensor using magneto-optical glass (Flint glass SF2), as shown in Fig. 17. The current sensor had a linear response range from 0 to 800 A [88].

Moreover, the temperature cross-sensitivity is the main difficulty for practical applications of optical fiber current sensors based on garnet crystal Faraday rotators. Qun Han et al*.* proposed a temperature compensation method. It compensated for the temperature dependence of the sensor by introducing static bias of a pair of toroidal permanent magnets. The results showed that the temperature relative errors of the sensor range are from ±8.0% to ±1.0% [89].

## Applications of optical fiber magnetic field sensors

Optical fiber magnetic field sensor technology has developed rapidly and has a wide range of application prospects, such as in optical current sensors, marine multidimensional detection, and national defense fields. In this section, we will focus on the applications of optical fiber current sensors, geomagnetic sensors, and quasi-distribution sensors.

### Optical fiber current sensors

In recent years, the electric power industry has grown rapidly and become an integral part of the world. To monitor the working status of electric power systems, safe, reliable and sensitive current sensors are urgently needed. Optical fiber current sensors have become a hot spot due to their low cost, small size, high accuracy, good insulation, and resistance to electromagnetic interference. Among the optical fiber current sensors with different working principles, optical fiber current sensors based on magneto-optical crystals have generated wider interest. These optical fiber current sensors are based on the Faraday magneto-optical effect, which essentially measures the strength of the magnetic field brought about by the change of current to determine the current magnitude.

In the 1970s, the Bonneville Power Administration in the United States manufactured one of the first instruments called the “Traser” optical fiber current sensor, which opened the research in this field [90]. In 1980, the first magneto-optical optical fiber current sensor appeared [91], which was then intensively studied by A. Papp et al*.* [92]. In the early 1990s, Toshiba used fiber optic current sensors in distribution network automation systems and has also developed fiber optic current sensors that can operate with 1000 kV systems. In 1995, ALSTOM developed a 525 kV fiber optic current sensor and successfully operated it in grid systems in the United States, the Netherlands, Belgium, and other countries. In 2003, a Canadian company, NxtPHASE, developed a fiber optic current sensor for 121–550 kV systems, whose performance exceeds the International Electrotechnical Commission (IEC) standard, with an accuracy level of current transformer of 0.2 S level.

**Fig. 18:**
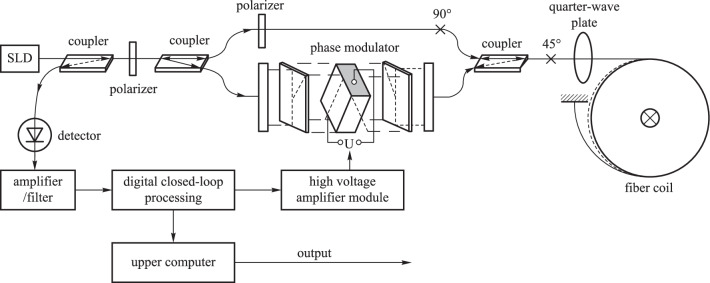
FOCT based on the spatial phase modulation method [94]. Copyright 2020, Qi Y. Reprinted with permission

Many new optical fiber current sensors have also been introduced in recent years. In 2017, Jiahui Han et al*.* proposed a temperature compensation method based on a dual fiber grating structure, with two fiber gratings connected to two identical magneto-strictive materials [93]. The authors experimentally showed that the method can reduce the effect of temperature and maintain good performance in the temperature range of 20–70 ℃. In 2020, Yuefeng Qi et al*.* proposed a common-line closed-loop optical fiber current sensor using spatially irreversible modulation method (see Fig. 18), which has improved the stability and sensitivity of optical fiber current sensors [94]. In 2020, Hongze Gao et al*.* employed a chiral birefringent photonic crystal fiber as a sensing coil to mitigate the residual linear birefringence in the optical fiber current sensor system. Simulation results showed that, by adjusting the chirality parameters, air filling ratio, lattice constant, and innermost pore diameter, the measurement accuracy could be improved by an order of magnitude [95].

### Geomagnetic sensors

The geomagnetic field is one of the fundamental research fields of the earth sciences. The geomagnetic field is present over the entire Earth and into near-Earth space. The measurement of the geomagnetic field in the natural environment has a wide range of applications in the fields of aerospace, earth sciences, resource exploration, national defense, transportation and communication, and earthquake forecasting. With the development of technology, more and more magnetic effects are utilized to make magnetic measurement devices, such as superconducting quantum interference devices (SQUIDs) and optically pumped magnetometers (OPMs). These devices have been widely used in modern geomagnetic measurement systems. Optical fiber magnetic field sensors are also widely used in magnetic measurement systems because of their good stability and high sensitivity.

In 1995, Frank Bucholtz et al*.* first proposed a multichannel optical fiber magnetometer system for measuring submarine geomagnetism [96]. The system consisted of eight fiber-optic vector magnetic field sensors and achieves stable and low-noise operation without frequent adjustments. In the same year, Coghill et al*.* reported an electrically passive optical fiber magnetometer for geomagnetic transient measurements. This sensor consisted of a metallic glass filament coated with a section of optical fiber, and its minimum detectable field recorded in the frequency response was 2.5 × 10^–8^ ${\mathrm{Gs}}/{\sqrt{\mathrm{Hz}}}$ at 2 kHz [97]. Later, in 2005, Xiaojun Zhou et al*.* bonded magnetostrictive materials to a fully conformal fiber to form a magnetic-optical phase change conversion and detected the phase change by a fully conformal fiber interferometer for geomagnetic field sensing experiments to achieve stable magnetic field detection [98]. The experimental results showed that the phase sensitivity of the fully bias-preserving optic al fiber magnetic field sensing system is 9 × 10^–6^ rad/nT, and the minimum measurable AC magnetic field signal shift could reach the nanometer level. The measured data are in good agreement with the theoretical values, indicating that the system can be used for high-sensitivity geomagnetic field measurements. After that, in 2018, Qilai Zhao et al*.* reported a 1083 nm single-frequency fiber laser with noise near the quantum limit and absolute frequency stability [99]. The low relative intensity noise at low frequencies and outstanding frequency stability of this fiber laser allows it to be applied to high-precision magnetometers.

In 2021, Jianjun Li et al*.* developed a miniature wide-range three-axis vector atomic magnetometer working in the spin-exchange relaxation-free regime by using lasers to detect the interaction between alkali metal atoms in the magnetic field. The sensor utilized a cylindrical gas chamber with a diameter and length of 10 mm, a polarization-maintaining fiber, and a multimode fiber to construct a single-beam configuration. A rotating field in the plane perpendicular to the laser propagation direction and another modulated field in the pump direction are generated by direct digital synthesis using a field-programmable gate array, allowing simultaneous measurement of the three components of the magnetic field. The sensitivity of the transverse magnetic field reached 350 ${\mathrm{fT}}/{\sqrt{\mathrm{Hz}}}$ and the sensitivity of the longitudinal field reached 3 ${\mathrm{pT}}/{\sqrt{\mathrm{Hz}}}$ in the dynamic range of 60 μT. The small size, wide range, and high sensitivity of this sensor make it particularly suitable for geomagnetic observation and space exploration [100].

### Quasi-distributed magnetic sensors

Quasi-distributed fiber magnetic field (QDFMF) sensing is an emerging optical fiber sensing technology based on light polarization and the magnetostriction effect. Compared with the traditional fiber optic magnetic field sensor, QDFMF sensor can be used to monitor the magnetic field intensity at multiple points. In addition, it has the advantages of fast response, low cost, corrosion resistance, high-temperature tolerance, good insulation performance, and resistance to electromagnetic interference. This allows QDFMF to have irreplaceable applications in national defense security, disaster warning, environmental monitoring, resource exploration, medical and health care, industrial manufacturing, and other fields [101, 102].

In 2005, a giant magneto-strictive magnetic field sensor based on a dual fiber Bragg grating was reported by Mingfan Li et al*.* [103]. The sensor system was simple in structure and had good linearity and sensitivity. The sensor system also achieved a quasi-distributed measurement of the magnetic field and obtained a sensitivity of 1.8 nm/Oe, but there were still problems such as temperature compensation of the magnetostriction of the super-magneto-strictive transducer and how to reduce the hysteresis effect. In 2011, Luca Palmieri et al*.* proposed a dual-cell magnetic field sensor based on polarization-sensitive reflectometry (polarization optical time-domain reflectometry (P-OTDR) and polarization optical frequency domain reflectometry (P-OFDR)) to achieve the measurement of static magnetic fields in a 1.5 T magnetic resonance imaging scanner [104]. In the following two years, they achieved magnetic field measurements by improving the arrangement of the fiber [105]. However, due to the insufficient coherence of the light source, the sensing distance range of this magnetic field sensor was less than 100 m. In 2014, a distributed fiber optic magnetic field sensor was reported by Ali Masoudi et al*.* This sensor measures the magnetic field by measuring the magneto-strictive induced strain of an optical fiber nickel wire, and the resolution of the sensor is 0.3 Gs in the magnetic field intensity range of 1–8 Gs [106]. However, the technique based on polarization optical time-domain reflectometry requires many complex modules, such as pulse modulation and high-speed detection, which leads to high costs of the system.

**Fig. 19:**
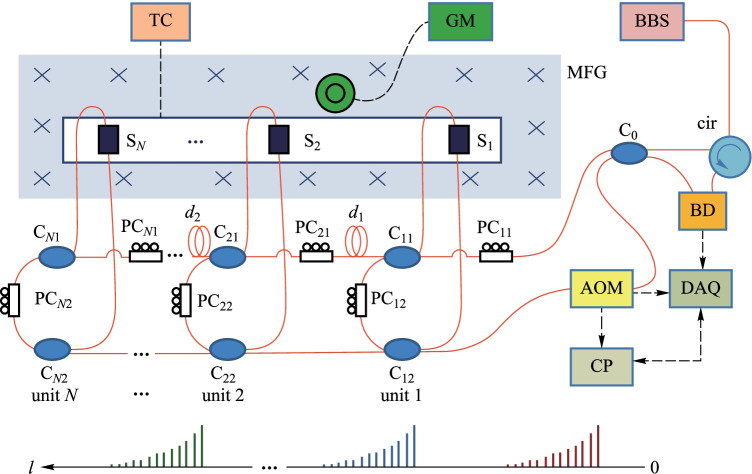
Configuration of the quasi-distributed magnetic field sensing system [107]. Copyright 2022, Elsevier B.V. Reproduction with permission

In 2022, Yiwen Ou et al*.* proposed a quasi-distributed fiber magnetic field sensing system based on the frequency-shifted interferometry fiber cavity ringdown (FSI-FCRD) technique (Fig. 19) [107]. The system uses the refractive index change of the magnetic fluid to realize magnetic field sensing and the absorption of evanescent waves by the laterally polished optical fiber to improve the sensitivity. The sensitivities of the two sensing units in the system are 6.7894 × 10^–4^ and 7.4980 × 10^–4^ dB/Oe, respectively.

Table 2 summarizes the performance of quasi-distributed magnetic field sensors, including sensing structure, sensing material, sensing sensitivity, and magnetic field measurement range.

**Table 2:** Performance parameters of quasi-distributed magnetic sensors

Sensing structure	Sensing materials	Sensitivity or intensity resolution^a^	Measurement range^a^	Reference
FBG	MS: Terfenol-D	18 pm/mT	20–70 mT	[103]
MZI	Nickel wire	0.03 mT	0.1–0.8 mT	[106]
FSI-FCRD	MF: Fe_3_O_4_	7.4980 × 10^−3^ dB/mT	0–25 mT	[107]

^a^The data units have been converted, and the conversion relationships are as follows: 1 T = 1000 mT, 1 mT = 10 Gs, 1 mT = 10 Oe, and 1 Oe = 1000/(4π) A/m.

## Conclusion and perspective

This paper introduces the basic principle and the latest development of optical fiber-based magnetic field sensors. The working principle and structural features of different fiber configurations, such as fiber gratings (FBG, LPFG, and TFBG), fiber-based interferometry (MZI, FPI, SI, and MI), and tailored fiber with evanescent field (tapered fiber, D-shape fiber, and U-shape) are discussed in detail. Magnetically sensitive materials can convert magnetic field change into the change of other physical parameters that can be detected by an optical fiber, such as refractive index, deformation, and stress, thus becoming indispensable in optical fiber magnetic field sensing. This paper also presents magnetically sensitive materials, including magnetic fluid materials, magnetic-strictive materials, and magneto-optical materials. In addition, the applications of fiber magnetic field sensors in current monitoring, geological detection, and distributed sensing systems are presented.

So far, optical fiber magnetic field sensors and magnetically sensitive materials involved in these sensors have some common problems. First, the industrialization of these optical fiber sensors needs to be improved. The problems of device packaging and the improvement of stability still need to be solved. Second, the sensitivity and anti-disturbance of optical fiber magnetic field sensors need to be improved. The sensitivity of optical fiber-based magnetometry is relatively low compared to other magnetometry technology, such as quantum optical magnetometry. Meanwhile, the tolerance to temperature change and environmental noise needs to be enhanced. Third, the distributed fiber optic sensing system already enabled precise measurements for temperature, vibration, acoustic wave, and deformation with high-spatial-resolution and high-sensitivity. While for the magnetic field sensing, only quasi-distributed measurements are currently possible. High-precision distributed sensing systems of the magnetic field have important applications in geological monitoring and resource exploration. In addition, with the development of fiber fabrication and material science, multi-functional multi-materials fiber also provides a new technology for an all-fiber magnetic field sensing system. The structured optical fiber and magnetically sensing materials can be combined to form integrated sensing fibers.
